# Pilot-scale steam explosion pretreatment with 2-naphthol to overcome high softwood recalcitrance

**DOI:** 10.1186/s13068-017-0816-y

**Published:** 2017-05-18

**Authors:** Thomas Pielhop, Janick Amgarten, Michael H. Studer, Philipp Rudolf von Rohr

**Affiliations:** 1 Institute of Process Engineering, ETH Zurich, Sonneggstrasse 3, 8092 Zurich, Switzerland; 2 School of Agricultural, Forest and Food Sciences, Bern University of Applied Sciences, Länggasse 85, 3052 Zollikofen, Switzerland

**Keywords:** Lignocellulose, Biomass, Softwood, Spruce, Steam explosion, Pretreatment, 2-Naphthol, Carbocation scavenger, Enzymatic hydrolysis, Biorefinery

## Abstract

**Background:**

Steam explosion pretreatment has been examined in many studies for enhancing the enzymatic digestibility of lignocellulosic biomass and is currently the most common pretreatment method in commercial biorefineries. It is however not effective for overcoming the extremely high recalcitrance of softwood to biochemical conversion. Recent fundamental research in small-scale liquid hot water pretreatment has shown, though, that the addition of a carbocation scavenger like 2-naphthol can prevent lignin repolymerization and thus enhance the enzymatic digestibility of softwood cellulose. This work studies the technical application potential of this approach in a larger steam explosion pilot plant for surmounting softwood recalcitrance.

**Results:**

The addition of 35.36 g 2-naphthol to the steam explosion pretreatment of 1.5 kg spruce wood chips allowed to considerably enhance the enzymatic cellulose digestibility. Different ways of adding the solid 2-naphthol to steam pretreatment were tested. Mixing with the biomass before pretreatment could enhance digestibility by up to 55% compared to control experiments. Impregnation of the biomass with 2-naphthol was yet more effective. Acetone and ethanol were tested to dissolve 2-naphthol and impregnate the biomass. The solvents were then removed again by evaporation before the pretreatment. The impregnation allowed to enhance digestibility by up to 179 and 192%, respectively. A comparison to prevalent acid-catalyzed steam explosion pretreatments for softwood revealed that the scavenger approach allows for obtaining exceptionally high yields in enzymatic hydrolysis. The biomass impregnation with 2-naphthol even renders a complete enzymatic cellulose conversion possible, which is remarkable for a softwood pretreatment not removing lignin. Steam pretreatment experiments without explosive decompression revealed that the enhancing effects of the explosion and the scavenger complement each other well. The explosion enhances the accessibility of the cellulose while the use of the scavenger reduces particularly the deactivation of enzymes.

**Conclusions:**

This is the first study to show that a carbocation scavenger in steam pretreatment can enhance the enzymatic digestibility of lignocellulosic biomass. The approach opens up a novel possibility for overcoming the high softwood recalcitrance in a process that does not require an acid catalyst or the removal of lignin from the biomass.

**Electronic supplementary material:**

The online version of this article (doi:10.1186/s13068-017-0816-y) contains supplementary material, which is available to authorized users.

## Background

The production of fuels and chemicals is greatly dependant on fossil resources. The demand for chemical resources will even increase with a growing world population and a rising global living standard and liquid fuels will still be needed in the foreseeable future [1]. Lignocellulose is an abundant biomass and therefore a promising source for the large-scale production of renewable fuels and organic chemicals. It is much more abundant than, e.g., seeds or fruits [1] and available at distinctly lower costs [2]. Although the processing of lignocellulosic biomass is more challenging, continuous improvements of its biochemical conversion have recently allowed the first commercial plants to enter production [3].

Softwood is the dominant lignocellulosic feedstock available in the Northern hemisphere and a potential source for fermentable carbohydrate in the United States, Canada, Scandinavia [4], South America [5], China [6], and Russia [7]. In the US for example, 30% of the harvested lignocellulosic biomass originates from forest biomass, thereof 60% account for softwood species [8, 9]. Woody raw materials offer flexible harvesting times, moderate transportation costs due to their high density and have a very low ash content compared to other types of lignocellulosic biomass, which facilitates their biochemical processing [5, 8]. Softwood is a promising feedstock being a fast and straight growing tree and having a particularly low pentose content. The fermentation of pentoses is still a challenge and thermo-chemical pretreatments easily decompose them to fermentation inhibitors such as furfural [10].

Softwoods are however much more refractory than hardwoods or agricultural residues and cost-effective hydrothermal/autohydrolysis methods such as steam pretreatment are not effective [5, 11]. Steam treatments can be enhanced by the addition of an acid catalyst like H_2_SO_4_ or SO_2_, which can increase process complexity and cost [12] but allows to obtain decent sugar yields from softwood [11]. Chemical delignification operations are very effective but also expensive, which is why they have not been adopted for woody biomass pretreatment [5]. Despite intensive research, no economically feasible process has been developed for bioconverting softwood so far [13–15]. Therefore, softwood is currently processed only via thermochemical technologies on a commercial scale [3], where its recalcitrance to biological processing does not play a role. Finding a method for reducing the high softwood recalcitrance via cost-effective pretreatment methods would therefore be of great benefit.

The difficulties in the bioconversion of softwood are attributed to its lignin type and content [16], though the reasons for its exceptional resistance are not well understood [4, 17]. Lignin is the most important but at the same time the most unknown factor in governing the digestibility of lignocellulose [18]. Elucidating the hindering effect of lignin is further complicated by the fact that the lignin content or its native structure is not necessarily the key factor, but also the way its structure is modified during pretreatment [19]. This is in accordance with our recent observation, revealing that the suppression of lignin repolymerization in softwood liquid hot water pretreatment can significantly enhance its enzymatic cellulose digestibility [20]. This can be achieved by the addition of a carbocation scavenger that reacts with lignin carbocations, which are formed in the lignin polymer due to the acidic conditions created by the release of acidic hemicellulose side groups. Those carbocations are supposed to be responsible for repolymerization reactions, as they can react with the electron-rich aromatic rings present in lignin [21, 22]. 2-Naphthol has proven as an effective carbocation scavenger in the liquid hot water pretreatment of softwood and allowed to increase glucose yields in enzymatic hydrolysis by up to 64%. It was revealed that the resulting less repolymerized lignin has a lower specific surface area with a reduced potential for the adsorption and deactivation of cellulolytic enzymes, which increases glucose yields. The 2-naphthol is nearly completely consumed in pretreatment and integrated into the lignin structure. Its residual concentration was shown to not hinder fermentation organisms such as *Saccharomyces cerevisiae* [20].

Stirred liquid hot water pretreatments do however not allow for high biomass loadings and thus demand a high energy input for heating up large amounts of water and for the downstream product purification, which is why they are not developed at commercial scale [12, 23]. In contrast, steam pretreatments allow for very high biomass loadings and are currently the dominating commercial pretreatment method [24]. Steam pretreatments additionally offer the possibility of a steam explosion. At the end of the steaming, the pressure is released abruptly and flash evaporation of condensed superheated water occurs. The evaporation of the “inner water” forces literally an explosion of the biomass and causes an extensive decrease of particle size. Steam explosion pretreatments can therefore deal with large biomass particles and reduce their size in an energy efficient way [25, 26], which is beneficial for the treatment of wood. Compared to agricultural biomass, the size reduction of woody biomass is particularly energy intensive [5]. Moreover, we have recently shown that the decrease of the particle size by the explosive decompression can distinctly enhance the enzymatic cellulose conversion of softwood [27].

In spite of the technical differences between liquid hot water and steam explosion pretreatment, the actual chemical changes introduced to the biomass and to the lignin are very similar [23, 28]. In steam pretreatment, the biomass and its capillary-like microporous structure is soaked with liquid water just as in liquid hot water pretreatment [29], and similar chemical processes can be assumed. So far however, carbocation scavengers have only been tested in stirred liquid hot water pretreatments and with biomass particles smaller than 1 mm in size. The application in a non-stirred steam explosion treatment and the use of larger wood chips is of much higher technical and economic relevance.

This faces however several potential difficulties, such as a thorough distribution of the scavenger additive in the pretreatment slurry, mass transfer limitations to the inner of the wood chips or an interference of the explosion with the scavenger effect. To address these open questions and to clarify the application potential of a carbocation scavenger, this work investigates the steam explosion pretreatment of spruce wood chips with 2-naphthol as a representative scavenger. In order to study the importance of mass transfer effects, two different ways of adding the solid scavenger were evaluated: mixing of the biomass with the solid catalyst and impregnation of the biomass with a 2-naphthol solution before pretreatment. The results are compared to state-of-the art acid-catalyzed steam explosion pretreatments for softwood.

## Methods

### Biomass

Spruce wood chips were prepared from a roughly 30-year-old tree, cut in summer 2014 in Biberist (canton of Solothurn, Switzerland) by chopping through a 30-mm screen. The fresh biomass had a dry matter of 46.2 ± 1.7%, and the composition was determined to be glucan 39.6 ± 0.9%, mannan 17.7 ± 1.6%, acid-soluble lignin (ASL) 5.22 ± 0.04%, acid-insoluble lignin (AIL) 29.0 ± 0.2%, and extractives 6.6 ± 0.4% (total 98.1%). After chopping, the wood chips were stored at 5 °C in sealed plastic bags during the experimental time of 4 months. For each experiment, 1.5 kg of wood chips was used.

### Addition of 2-naphthol

In experiments with scavenger, 35.36 g of 2-naphthol (Sigma-Aldrich, ≥98%) were added to the biomass which corresponds to a concentration of 0.205 mol mol^−1^ lignin C_9_ unit (assumed molecular weight of C_9_ unit 185 g mol^−1^). In one experimental set, 2-naphthol was added by blending the wood chips with the 2-naphthol flakes (≤0.5 mm). Blending was carried out by hand in a 5-l beaker to uniformly mix wood chips and 2-naphthol flakes before filling the mixture into the steam gun reactor. In another experimental set, the wood chips were impregnated with the 2-naphthol. Therefore, it was dissolved in 5 l of either acetone or ethanol to completely cover the biomass with the impregnation solution. The solvent was then allowed to evaporate at room temperature in a vented fume hood with frequent mixing to assure even impregnation. The evaporation of the acetone and ethanol impregnation solution lasted 1 and 3 days, respectively. Afterwards, the wood chips were further air-dried for 4 weeks to ensure complete removal of the solvent from the biomass.

### Steam gun and pretreatment

The pretreatment reactor is made of stainless steel with a volume of 5.8 l (DN100, i.e., 114.3 mm inner Ø, 700 mm inner height). Steam of 32 bar (absolute pressure) is injected to reach the target pressure in the reactor. Entrapped air is removed by an exhaust valve at the top of the reactor, so that the steam saturation temperature corresponding to the pressure setpoint is reached. A ball valve at the bottom of the reactor allows for the discharge of the biomass into the blow tank. The valve is driven by a pneumatic actuator operated at 10 bar air pressure to ensure a fast opening and depressurization as needed for steam explosion. Alternatively, the pressure can also be released via a hand valve at the bottom of the reactor into a secondary tank. More details on the steam explosion system (steam generation and injection, removal of entrapped air, construction details and schematic drawing) can be found elsewhere [27].

Steam treatments were carried out with an absolute steam pressure in the reactor of 31 bar, corresponding to a saturated steam temperature of 235 °C. After a pretreatment time of 2.5, 5, 10 or 20 min, the biomass was exploded/discharged into the blow tank. In order to better elucidate the interaction of the explosion and the scavenger effect, pretreatments with admixed 2-naphthol were also carried out without explosive decompression. Therefore, the pressure was bled off via the hand valve into the secondary tank at the end of the pretreatment before the biomass was discharged with a reduced pressure of 2.5 bar into the blow tank. This overpressure of 2.5 bar was necessary for emptying the gun and experiments which were conducted with this low explosion pressure are referred to as experiments “without explosion” in this work. The slurry obtained in all pretreatments was weighed and then filtered recording weight and pH of the filtrate. The biomass filter cake was not washed and its dry matter content was determined in duplicate. More details on the pretreatment procedure (preheating of reactor, time measurement, experiments without explosion) can be found elsewhere [27].

An overview of the experimental conditions and pretreatment severities is shown in Table 1. The pretreatment severity was defined as Eq. (1).

**Table 1:** Overview of pretreatment experiments and experimental conditions

2-Naphthol addition	*T*/°C	*t*/min	log*R*_0_/−	Δ*p* explosion/bar
–	235	2.5	4.4	30
–	235	5	4.7	30
–	235	10	5.0	30
–	235	15	5.2	30
–	235	20	5.3	30
Mixing	235	2.5	4.4	30
Mixing	235	5	4.7	30
Mixing	235	10	5.0	30
Mixing	235	15	5.2	30
Mixing	235	20	5.3	30
Mixing	235	2.5	4.4	2.5*
Mixing	235	5	4.7	2.5*
Mixing	235	10	5.0	2.5*
Mixing	235	15	5.2	2.5*
Mixing	235	20	5.3	2.5*
Acetone impregnation	235	2.5	4.4	30
Acetone impregnation	235	5	4.7	30
Acetone impregnation	235	10	5.0	30
Acetone impregnation	235	15	5.2	30
Acetone impregnation	235	20	5.3	30
Ethanol impregnation	235	2.5	4.4	30
Ethanol impregnation	235	5	4.7	30
Ethanol impregnation	235	10	5.0	30
Ethanol impregnation	235	15	5.2	30
Ethanol impregnation	235	20	5.3	30

* Experiments with a Δ*p* of 2.5 bar are referred to as experiments “without explosion”

1 $$R_{0} = t \cdot e^{{\frac{T - 100}{14.75}}} ,$$

where *t* is the pretreatment time in minutes and *T* the pretreatment temperature in degrees Celsius [30].

### Biomass analysis

The dry matter and the composition (glucan, mannan, AIL, ASL, extractives) of the raw and pretreated biomass as well as the sugar contents (glucose, sum of hemicellulosic sugars) in the pretreatment liquor were determined by the methods published by the National Renewable Energy Laboratory (NREL) [31–35]. The pretreated and dried biomass was however pulverized by pestling before compositional analysis. All biomass and pretreatment liquor analyses were done in triplicate and duplicate, respectively, and single standard deviations are reported with the mean in this work.

To study the influence of 2-naphthol on the particle size reducing effect of the explosion, the particle size distribution of biomass pretreated without additive and with admixed 2-naphthol was analyzed by wet sieve analysis as described by Pielhop et al. [27].

### Enzymatic hydrolysis

The pretreated biomass underwent enzymatic hydrolysis according to the NREL standard procedure with a cellulose concentration of 1% w/w [36]. The following changes were made: sodium azide at a final concentration of 0.01 g l^−1^ was used instead of antibiotics and the pH was adjusted to 5.0 (0.05 mol l^−1^ sodium citrate buffer after sample preparation). 10-ml samples were prepared in 20-ml scintillation vials (VWR, USA). Due to the larger particle size, biomass that had been pretreated without explosion was prepared as 150-ml samples in 250-ml laboratory glass bottles (Schott, Germany). Accellerase 1500 (Genencor; lot number 4901298419), with an activity of 26 filter paper units (FPU) ml^−1^ measured according to the NREL method [37] was used with final concentrations of 15, 30, and 60 FPU g^−1^ cellulose in the sample preparation. Samples were incubated in a shaker (Multitron; Infors-HT) with a shaking throw of 25 mm at 50 °C and 210 rpm for 120 h and then analyzed for sugars in the supernatant. All hydrolysis experiments were carried out in triplicate and single standard deviations are reported with the mean of the cellulose digestibility.

### Sugar analysis

Sugar analysis by HPLC (high-performance liquid chromatography) was performed as described in the NREL procedure [32] using a Waters 2695 Separation module equipped with a Waters 410 differential refractometer and a Bio-Rad Aminex HPX-87H column.

### Yield calculations

The enzymatic cellulose digestibility was calculated as defined in Eq. (2).

2 $${\text{Digestibility}}_{\text{Cellulose}} = \frac{m_{{\text{Glucose, EH sample}}} \cdot 0.9}{{m_{{{\text{Glucan, EH sample}}}} }},$$

where *m*_Glucose,EH sample_ is the mass of glucose released during the enzymatic hydrolysis experiment and *m*_Glucan,EH sample_ is the mass of glucan added to the hydrolysis experiment with the pretreated biomass. The factor 0.9 accounts for the conversion of the anhydrous polymer to the monosaccharide. The hemicellulose (mannan) digestibility was calculated analogously.

The corresponding glucose yield that can be obtained from the recovered pretreated biomass by enzymatic hydrolysis was calculated as defined in Eq. (3).

3 $${\text{Yield}}_{\text{Glucose,EH}} = {\text{Digestibility}}_{\text{Cellulose}} \cdot \frac{{m_{\text{Glucan, Recovered}} }}{{m_{\text{Glucan,Feedstock}} }},$$

where *m*_Glucan,Recovered_ is the mass of glucan recovered with the pretreated biomass and *m*_Glucan,Feedstock_ is the mass of glucan added to pretreatment with the feedstock. In that way, losses due to glucan degradation in pretreatment and re-collecting of the pretreated biomass are accounted for. The enzymatic hydrolysis yield from hemicellulose was calculated analogously.

The yield of glucose released to the pretreatment liquor was calculated as defined in Eq. (4).

4 $${\text{Yield}}_{\text{Glucose,Pretreatment liquor}} = \frac{{m_{\text{Glucose,Pretreatment liquor}} \cdot 0.9}}{{m_{\text{Glucan,Feedstock}} }},$$

where *m*_Glucose,Pretreatment liquor_ is the mass of glucose in the recovered pretreatment liquor. The yield of hemicellulosic sugars (represented as mannose) in the pretreatment liquor was calculated analogously.

The total sugar yield summing up the glucose and hemicellulosic sugar yields from pretreatment and enzymatic hydrolysis was calculated as defined in Eq. (5).

5 $$\begin{aligned} {\text{Yield}}_{\text{Sugar,Total}} & = \frac{{ ( {\text{Yield}}_{\text{Glucose,Pretreatment liquor}} + {\text{Yield}}_{\text{Glucose,EH}} )\cdot m_{\text{Glucan,Feedstock}} }}{{m_{\text{Glucan,Feedstock}} + m_{\text{Mannan,Feedstock}} }} \\ & \quad + \frac{{ ( {\text{Yield}}_{\text{Mannose,Pretreatment liquor}} + {\text{Yield}}_{\text{Mannose,EH}} )\cdot m_{\text{Mannan,Feedstock}} }}{{m_{\text{Glucan,Feedstock}} + m_{\text{Mannan,Feedstock}} }}. \\ \end{aligned}$$

## Results and discussion

The steam pretreatments were carried out at a high temperature of 235 °C to enhance softwood cellulose digestibility as much as possible. High pretreatment temperatures and low residence times were shown to be favorable for the steam explosion pretreatment of spruce [38]. Moreover, a sensitivity analysis of temperatures between 195 and 225 °C in the liquid hot water pretreatment of spruce with 2-naphthol has shown that cellulose digestibilities peak at the highest temperature of 225 °C [39]. High temperatures do also correspond to a high steam pressure in the reactor, leading to an enhanced effect of the explosion on digestibility [27]. The pretreatments at high temperature did not lead to severe cellulose degradation. In average, 91.5% of the cellulose was recovered after pretreatment and the highest severity (log*R*_0_ = 5.3, *t* = 20 min) still allowed for an average recovery of 88.9% (Additional file 1: Table S1).

2-Naphthol was added at a concentration of 0.205 mol mol^−1^ lignin C_9_ unit, which has been shown to considerably enhance softwood digestibility in liquid hot water pretreatment [20, 40].

### Mixing of 2-naphthol and wood chips

**Fig. 1:**
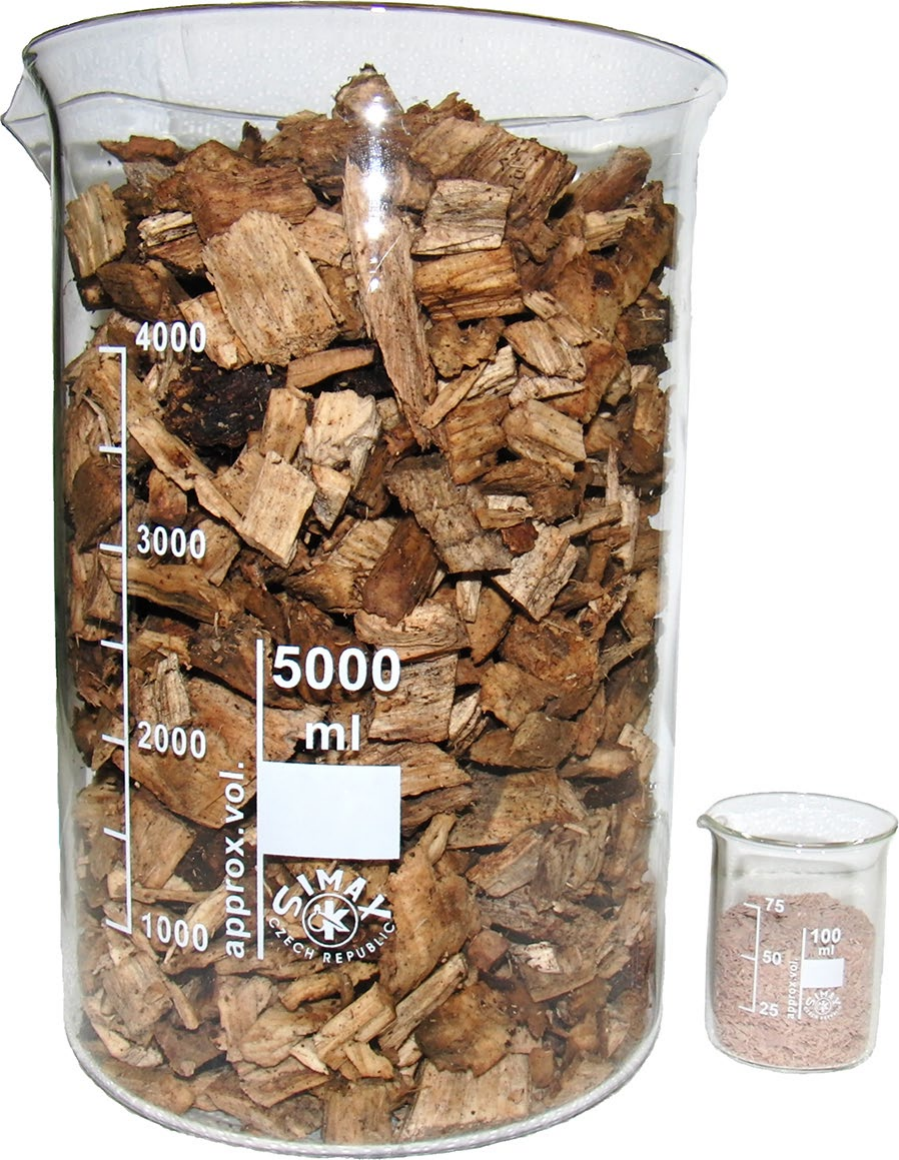
Representation showing the amounts of spruce wood chips (1.5 kg) and 2-naphthol (35.36 g) that were used in the experiments. The 2-naphthol was added by mixing of the solid flakes with the wood chips or by impregnation of the biomass prior to pretreatment

At ambient conditions, 2-naphthol is a solid in the form of a powder or flakes (compare Fig. 1) and the simplest way of adding it to a steam pretreatment is by mixing with the biomass before steaming. The flakes in the mix stick to the moist wood chips so that the blend is not demixed when filling the reactor. 2-Naphthol has a very low water solubility of 0.75 g l^−1^ at 25 °C, which increases however exponentially with rising temperature [41]. The moisture content of the biomass alone would be sufficient for dissolving all added 2-naphthol at a temperature of 127 °C,^1^ showing that the experiments at 235 °C were carried out well above the solubility limit of 2-naphthol. 2-Naphthol can also make up a noticeable proportion in the vapor phase of the two component system water-2-naphthol, with a solubility limit of 3.1% w/w in steam at 235 °C and 31 bar.^2^ However due to the comparatively low steam density, at most 2.4 g of the used 35.36 g of 2-naphthol may be found in the vapor phase of the 5.8 l reactor volume. Therefore, in order to develop its desired effect on lignin, the bulk of the 2-naphthol needs to get dissolved in the condensed steam and biomass moisture and then be distributed via diffusion in the liquid phase. Furthermore, the 2-naphthol must penetrate into the inner of the wood chips by diffusion.

**Fig. 2:**
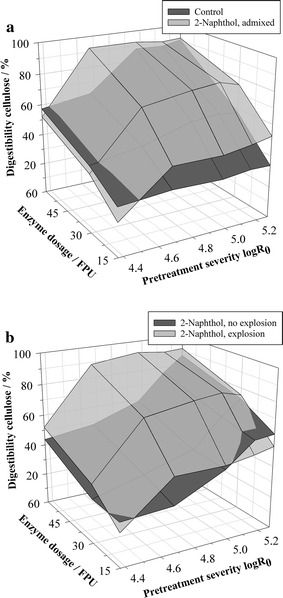
Influence of 2-naphthol admixture to steam pretreatment on enzymatic cellulose digestibility. **a** Steam explosion pretreatment without additive (control) and with admixed 2-naphthol. **b** Steam explosion pretreatment with admixed 2-naphthol, carried out with and without explosive decompression. Digestibility is expressed as glucose yield of pretreated biomass content. Pretreatment conditions: *T* = 235 °C, *t* = 2.5–20 min, Δ*p* explosion = 30 bar, 1.5 kg wood chips, 35.36 g 2-naphthol; hydrolysis conditions: 1% w/w cellulose, 15/30/60 FPU g^−1^ cellulose

In spite of those potential mass transfer limitations, the admixed scavenger is indeed effective in the steam pretreatment and enhances the cellulose digestibility. The enzymatic cellulose conversions of spruce wood chips that were steam explosion pretreated with 2-naphthol are shown in Fig. 2a. For comparison, results from the steam explosion experiments without additive are represented as control. Results are shown for different pretreatment severities (log*R*_0_ = 4.4, 4.7, 5.0, 5.2, and 5.3) and enzyme dosages of 15, 30, and 60 FPU g^−1^ cellulose (numerical values of cellulose digestibilities and standard deviations are provided in Additional file 1: Table S2). The highest enhancing effect of the scavenger can be found for the lowest enzyme dosage of 15 FPU g^−1^ cellulose, where it could improve the digestibility by up to 55% relatively compared to the control (log*R*_0_ = 5.3). For higher enzyme dosages, the 2-naphthol develops a good effect particularly at medium severities. It can improve the digestibility by up to 50% (log*R*_0_ = 4.7, 30 FPU g^−1^ cellulose) and allows for an almost complete cellulose conversion at lower severities than the control (log*R*_0_ = 4.7, 60 FPU g^−1^ cellulose). Those experiments show for the first time that a carbocation scavenger can enhance the steam pretreatment of lignocellulosic biomass.

The admixed 2-naphthol had no influence on the AIL, cellulose, and hemicellulose content of the pretreated biomass (Fig. 4c, d) nor on the pH of the pretreatment liquor (Additional file 1: Figure S1). It did however increase the measured ASL content in the pretreated biomass by up to 75% (Fig. 4c). The aromatic 2-naphthol structure is integrated into the lignin molecule forming a covalent bond, which is supposed to increase UV-absorption in the ASL measurement. In addition, the suppression of lignin repolymerization with 2-naphthol leads to a lower molecular weight, which can increase lignin solubility and thus lead to an actual increase of the ASL content. The observed effect on the ASL content is similar as it has been reported previously in liquid hot water pretreatments with 2-naphthol [20], further confirming that 2-naphthol is effectively applicable to a steam pretreatment, too.

A sieving analysis of the pretreated biomass showed that the 2-naphthol did not have any influence on the particle size of the exploded biomass (results not shown). It can thus be assumed that it did not influence cellulose digestibility by affecting the biomass particle size, e.g., by weakening the lignocellulose structure and leading to an increased defibration/pulverization effect of the explosion.

In order to further elucidate the coaction of the scavenger and the explosion effect on cellulose digestibility, experiments with 2-naphthol were also conducted without explosive decompression. The corresponding results in enzymatic hydrolysis are shown in Fig. 2b and compared to the 2-naphthol pretreatments with explosion. The explosion did in most cases distinctly improve the pretreatment and could enhance cellulose conversion by up to 97% relatively compared to the non-exploded samples (log*R*_0_ = 4.7, 30 FPU g^−1^ cellulose). The enhancement can be attributed to the reduction of the biomass particle size, which increases the accessibility of cellulose to the enzymes [27]. This perception is supported by the observation that in experiments without explosion, the enzyme dosage did practically not influence glucose yields. The access to the cellulose seems to be restricted so that increasing the enzyme dosage does not allow for higher sugar yields. The pretreatment can therefore greatly profit from the synergistic effects of the explosion and the scavenger.

Surprisingly, very high severities lead to a fairly accessible cellulose also in the pretreatments without explosion. At the highest severity of log*R*_0_ = 5.3, a cellulose digestibility of up to 86% can be reached. Such a high cellulose accessibility has not been observed in similar steaming experiments without explosion that were performed without scavenger [27]. The mechanism how the combination of a carbocation scavenger and a high pretreatment severity can increase cellulose accessibility has still to be elucidated. It has already been observed, though, that intense lignin repolymerization may also shield the cellulose from enzymatic attack, which can be prevented by the use of a scavenger [40].

### Impregnation of biomass with 2-naphthol

In another experimental set, the scavenger was added by impregnation of the biomass. In that way, the 2-naphthol has penetrated to the inner of the wood chips before the pretreatment and potential mass transfer limitations and maldistribution can be reduced. In particular, the 2-naphthol does not need to be distributed by diffusion in the liquid, which might also involve pore diffusion limitations when penetrating into the solid wood chips. The 2-naphthol is distributed more uniformly within the biomass and its “effective” concentration in the biomass may even be higher compared to the mixing experiments. The extraction of impregnated 2-naphthol from the chips by the steam condensate is diffusion-controlled, bringing about a higher concentration in the biomass especially at the beginning of the pretreatment.

2-Naphthol impregnation of the biomass was tested with acetone and ethanol as solvent. The solvents were removed by evaporation during the impregnation and the following air drying process and did not reach the pretreatment. Due to ambient humidity, air-dried biomass holds a water content of usually below 10% [31] and the water content of the wood chips at the end of the drying was determined to be 7.7 ± 0.8%. It is noted that the impregnation process may influence the distribution of extractives in the biomass prior to the pretreatment, which then removes the major part of the extractives from softwood biomass [42].

**Fig. 3:**
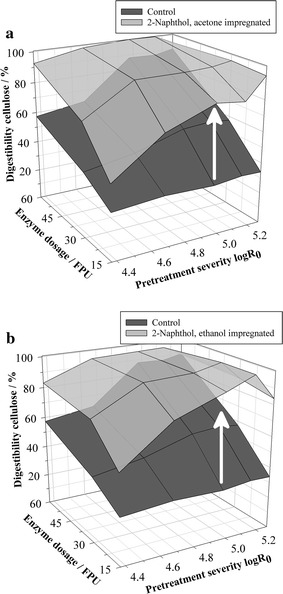
Influence of biomass impregnation with 2-naphthol before steam explosion pretreatment on enzymatic cellulose digestibility. **a** Steam explosion pretreatment without additive (control) and with 2-naphthol impregnation using acetone as solvent. **b** Steam explosion pretreatment without additive (control) and with 2-naphthol impregnation using ethanol as solvent. Solvents were removed by evaporation before the pretreatment. Digestibility is expressed as glucose yield of pretreated biomass content. Pretreatment conditions: *T* = 235 °C, *t* = 2.5–20 min, Δ*p* explosion = 30 bar, 1.5 kg wood chips, 35.36 g 2-naphthol; hydrolysis conditions: 1% w/w cellulose, 15/30/60 FPU g^−1^ cellulose

Figure 3a shows the cellulose conversions in the enzymatic hydrolysis of steam exploded biomass that had been 2-naphthol-impregnated using acetone. Results are shown for different pretreatment severities (log*R*_0_ = 4.4, 4.7, 5.0, 5.2, and 5.3) and enzyme dosages of 15, 30, and 60 FPU g^−1^ cellulose (numerical values of cellulose digestibilities and standard deviations are provided in Additional file 1: Table S2). It can be seen that the impregnation with 2-naphthol leads to an outstanding enhancement of the digestibility in comparison to the control without additive. The effect is particularly high at the lowest enzyme dosage of 15 FPU g^−1^ cellulose, where the digestibility could be enhanced by up to 179% relatively compared to the control (log*R*_0_ = 5.3). Those results are promising for overcoming the high recalcitrance of softwood, since enzymes are a major factor of biorefinery raw material costs and enzyme dosages of 15 FPU g^−1^ cellulose are frequently assumed for assessing the economics of biorefinery operations [43, 44]. Higher enzyme dosages do even render a complete cellulose conversion of effectively 100% possible. To the best of the authors’ knowledge, this has to date only been observed in an organosolv pretreatment of softwood chips which combines a steam pretreatment and ethanol extraction of lignin with H_2_SO_4_ as catalyst [45].

In contrast to adding the scavenger by mixing (compare Fig. 2a), the 2-naphthol impregnation with acetone allowed for a nearly complete cellulose conversion already at the lowest severity (log*R*_0_ = 4.4, 60 FPU g^−1^ cellulose). A lower severity translates to a lower pretreatment time, revealing that the scavenger added by impregnation develops its effect faster. Mass transfer effects probably delay the scavenger effect when added by mixing. Impregnation of the biomass presumably allows to prevent lignin repolymerization from the very beginning of the pretreatment and for lower pretreatment severities in order to obtain a highly digestible cellulose.

Acetone has a high vapor pressure, which facilitates its removal/recycling before the pretreatment in a technical evaporation process. The suitability of other solvents is however of interest, too. For instance, ethanol dissolves 2-naphthol as well and can be produced itself in a cellulosic ethanol process. Such a solvent should therefore not interfere with the downstream processing even if not completely removed from the biomass before pretreatment, which however was not the case in this study. Figure 3b shows the cellulose conversions in the enzymatic hydrolysis of steam exploded biomass that had been 2-naphthol-impregnated using ethanol. The results show that the impregnation with ethanol works also very efficiently and the cellulose digestibility was enhanced by up to 192% relatively compared to the control (log*R*_0_ = 5.2, 15 FPU g^−1^ cellulose). The pattern of the enhancing effect is very similar compared to the impregnation experiments with acetone, showing that the approach works equally with different solvents.

**Fig. 4:**
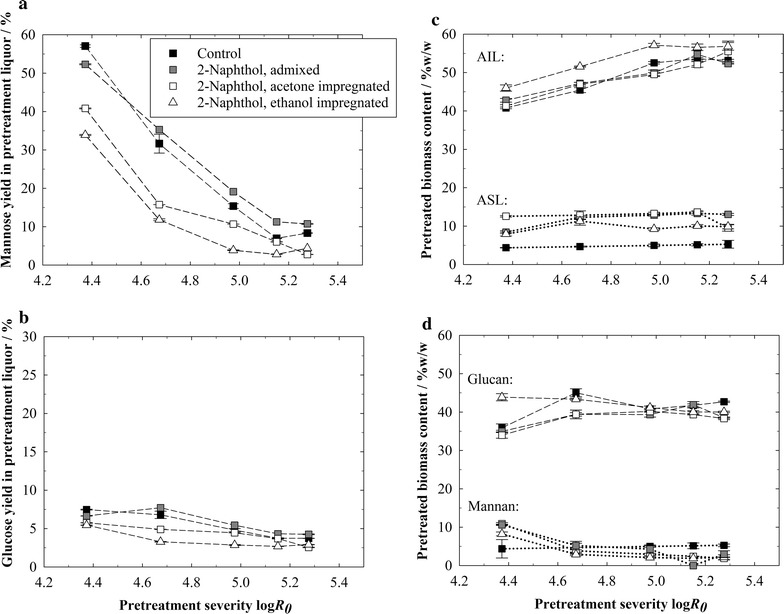
Sugar yields in pretreatment liquor (**a**, **b**) and biomass composition (**c**, **d**) after steam explosion pretreatments without additive and with 2-naphthol addition by mixing and impregnation. Sugar yields are expressed as % of raw biomass content. *AIL* acid-insoluble lignin, *ASL* acid-soluble lignin. Pretreatment conditions: *T* = 235 °C, *t* = 2.5–20 min, Δ*p* explosion = 30 bar, 1.5 kg wood chips, 35.36 g 2-naphthol

The 2-naphthol impregnation with acetone and ethanol increases the ASL content of the pretreated biomass compared to the control (Fig. 4c), due to the same reasons as described for the admixing experiments. The AIL content in the biomass pretreated with 2-naphthol is similar compared to the control (Fig. 4c). It is pointed out that the scavenger process does not remove lignin from the biomass and increases the biomass digestibility in that way.

Dissolved hemicellulosic sugars can considerably degrade in the pretreatment liquor, especially at high severities (Fig. 4a). The mannose and glucose yields in the pretreatment liquor were decreased by the 2-naphthol impregnation of the biomass, both with acetone and ethanol (Fig. 4a, b). A possible explanation is that the scavenger use might enhance the disintegration of the hemicellulose–lignin structure, so that hemicellulosic sugars dissolve and degrade faster in the acidic liquor. This may also explain why mannose yields in the following enzymatic hydrolysis were lower compared to the control and compared to experiments with admixed 2-naphthol, especially at higher enzyme dosages (Additional file 1: Table S2). Further elucidation of this effect is necessary, though. The pH of the pretreatment liquor was not influenced by the biomass impregnation with 2-naphthol (Additional file 1: Figure S1).

### Comparison of softwood steam explosion pretreatments

The potential of the scavenger process can be assessed by comparing it to other steam explosion pretreatments of softwood. Acid-catalyzed steam explosion is the most common steam pretreatment for woody biomass [5] and has been described as the most suitable method for softwood in a review by Galbe and Zacchi [11]. The review also compared the optimized conditions of one- and two-stage acidic steam explosion pretreatments for achieving the highest total sugar yield from cellulose and hemicellulose. It found that two-stage pretreatments allow for distinctly higher sugar yields from hemicellulose compared to one-stage pretreatments. In a two-stage pretreatment, a mild first stage dissolves and recovers the hemicellulosic sugars before a second harsher stage. Due to the additional sugar recovery, two-stage pretreatments also rank first in total sugar yields.

A similar ranking is reproduced here in Table 2, including several additional studies [38, 46, 47] and also the scavenger pretreatments of this work. Results are shown for the control, the scavenger addition by mixing and the scavenger addition by impregnation, for conditions that allowed for the highest total sugar yield (all experimental cellulose digestibilities and sugar yields can be found in Additional file 1: Table S2).

Although the varying process conditions of the different studies such as catalyst concentration, pretreatment severity or biomass particle size (see Table 2) make an in depth comparison difficult, mainstream trends can be observed. The selected studies use spruce or pine as feedstock, which were shown to have a very similar behavior of recalcitrance and can equally profit from the addition of 2-naphthol to pretreatment [40]. The studies employ a solid content in enzymatic hydrolysis of 2–3% w/w, which is in good agreement with the solid content in the hydrolysis of the 2-naphthol experiments (2.2–3.0% w/w) and allows for a good comparability. Literature studies using enzyme doses as low as 15 FPU g^−1^ cellulose could not be found, disclosing again the high recalcitrance of softwood biomass.

**Table 2:** Comparison of steam explosion pretreatment studies for softwood

References	Stages	Additive/catalyst	*T*/°C	*t*/min	Feedstock	Washing after pret.	FPU g^−1^ cellulose	Glucose EH/%	Mannose EH/%	Glucose P/%	Mannose P/%	Total sugar/%
	1	–	235	5	Spruce chips <30 mm	No	15	31	0	7	32	36
							30	45	2			46
							60	58	3			63
	1	2-Naphthol admixed	235	5	Spruce chips <30 mm	No	15	44	4	8	35	48
							30	67	2			63
							60	86	13			80
	1	2-Naphthol, acetone imp.	235	5	Spruce chips <30 mm	No	15	67	1	5	16	55
							30	93	2			70
							60	98	2			73
	1	2-Naphthol, ethanol imp.	235	5	Spruce chips <30 mm	No	15	81	1	3	12	64
							30	95	2			74
							60	95	5			75
Ballesteros et al. [46]	1	–	210	4	Pine chips 8–12 mm	Yes	32	32			60	41
Fang et al. [38]	1	H_2_SO_4_^a^	200		Spruce chips 1–20 mm	Yes	39	26		5	10	42
Stenberg et al. [11, 52]	1	SO_2_^b^	210	5.5	Spruce chips 2.2–10 mm	Yes	30	58		13	52	66
Tengborg et al. [11, 48]	1	H_2_SO_4_^c^	210	1	Spruce chips <30 mm	Yes	30	39		33	55	67
Monavari et al. [47]	1	SO_2_^d^	200	20	Spruce chips 5–6 mm	Yes	28	51	7	15	78	71
Nguyen et al. [50]	1	H_2_SO_4_^e^	215	1.7	Fir + Pine chips <12.7 mm	Yes	60	50		30	65	75
Söderström et al. [11, 51]	2	H_2_SO_4_^f^	180/200	10/2	Spruce sawdust	Yes	30	36		41	96	77
Söderström et al. [11, 55]	2	SO_2_^g^	190/220	2/5	Spruce chips 2.2–10 mm	Yes	30	45		35	95	80
Nguyen et al. [50]	2	H_2_SO_4_^h^	180/210	4/1.5	Fir + Pine chips <12.7 mm	Yes	60	25		57	84	82

Shown are the conditions for the highest total sugar yield. Yields are expressed as % of the raw biomass content

*EH* yield in enzymatic hydrolysis (accounting for cellulose recovery from pretreatment), *P* yield in pretreatment liquor, *Mannose* hemicellulosic sugars excluding glucose

^a^Impregnated with 0.6% w/w H_2_SO_4_ based on wood dry matter

^b^Impregnated with 3.5% w/w gaseous SO_2_ based on wood dry matter

^c^2.4% w/w H_2_SO_4_ in pretreatment liquid

^d^Impregnated with 2.5% w/w gaseous SO_2_ based on water moisture

^e^0.43% w/w H_2_SO_4_ in pretreatment liquid

^f^1st stage: impregnated with 0.5% w/w H_2_SO_4_ based on wood moisture; 2nd stage: impregnated with 2% w/w H_2_SO_4_ based on wood moisture

^g^Impregnated with 3% w/w gaseous SO_2_ based on wood moisture

^h^1st stage: 0.43% w/w H_2_SO_4_ in pretreatment liquid; 2nd stage: 2.5% w/w H_2_SO_4_ in pretreatment liquid

The ranking demonstrates that a mere steam pretreatment is not very effective for softwood. When comparing studies with an enzyme dosage of ~30 FPU g^−1^ cellulose, the uncatalyzed steam explosion pretreatment of pine wood chips carried out by Ballesteros et al. [46] shows the lowest total sugar yield of 41%. Only 32% of the glucose from the biomass can be released in the enzymatic hydrolysis. Increasing the pretreatment severity can allow for somewhat higher yields in the enzymatic conversion, however on the expense of a lower hemicellulosic sugar yield in the pretreatment liquor. The more severe pretreatment in the control experiments of the present work allowed for a yield of 45% in enzymatic saccharification, while practically reducing hemicellulosic sugar yields by half compared to the study of Ballesteros et al. [46].

**Fig. 5:**
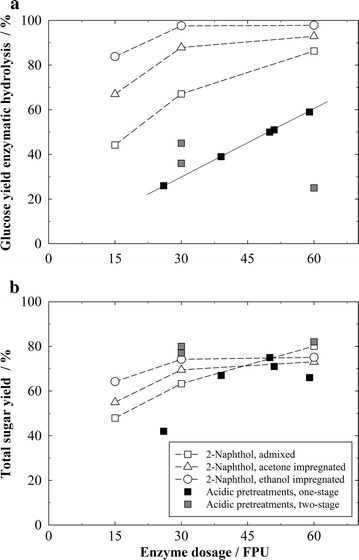
Comparison of 2-naphthol steam explosion pretreatments with acid-catalyzed steam explosion pretreatment studies [38, 47, 48, 50–52, 55] for softwood. Shown are the glucose yields in enzymatic cellulose hydrolysis (**a**) and the total sugar yields from the combined operations of pretreatment and enzymatic hydrolysis (**b**). Yields are expressed as % of raw biomass content and account for cellulose recovery from pretreatment. Pretreatment conditions for the 2-naphthol experiments: *T* = 235 °C, *t* = 5 min, Δ*p* explosion = 30 bar, 1.5 kg wood chips, 35.36 g 2-naphthol

In order to render higher sugar yields from softwood possible, an additive such as an acid catalyst or carbocation scavenger is very advantageous. For a better comparability of the different processes with additive, their glucose yields obtained in enzymatic hydrolysis and their total sugar yield are shown as a function of the used enzyme dosage in Fig. 5. Interestingly, the enzymatic hydrolysis yields of the acid-catalyzed one-stage pretreatments correlate linearly with the enzyme dosage, as illustrated in Fig. 5a. The glucose yields obtained in enzymatic hydrolysis with the scavenger processes are distinctly higher compared to all catalyzed pretreatments, up to four times higher yields are reached. Still, the acid-catalyzed processes achieve similar total sugar yields since they dissolve a large part of the cellulose in the pretreatment liquor. This is in particular true for the two-stage treatments, which dissolve up to 57% cellulose (compare Table 2). However, the designation of such processes as a “pretreatment” for biotechnological cellulose conversion is very floating. The process characteristics start to be determined by the acid cellulose hydrolysis and not by the biotechnological conversion anymore. Acid cellulose hydrolysis suffers however from sugar degradation and byproduct formation due to the sugar dissolution in the pretreatment liquor. In addition, costs for acid and neutralization chemicals, expensive reactor materials as well as environmental issues for e.g., properly disposing the produced salt from neutralization play a role. This is why enzymatic cellulose conversion is seen as a more viable strategy and the majority of the current commercial processes that convert lignocellulose to sugars employ enzymes [2, 7, 23]. Thus, the comparison reveals the high potential of a scavenger pretreatment for softwood. The process allows for exceptionally high glucose yields from enzymatic conversion, especially if the scavenger is added by impregnation, and outperforms every process in the total glucose yield. It also exceeds the total sugar yield of most acid-catalyzed one-stage pretreatments and even reaches the total sugar yields of two-stage pretreatments.

Those high yields are remarkable as the pretreatment process has not yet been optimized. It should also be emphasized that the enzymatic hydrolysis was performed with unwashed biomass, which was implemented in all other processes to reduce byproduct inhibition. Further, the wood chips had the largest size in the comparison along with the study of Tengborg et al. [48], which may negatively impact enzymatic cellulose conversion [27]. Presently, the total sugar yield in the scavenger process is limited by the degradation of hemicellulosic sugars in a single-stage pretreatment with high severity. Efforts to implement a two-stage pretreatment should allow for exceptionally high total sugar yields from softwood.

A recent theoretical assessment of glucose production technologies from softwood has shown that the scavenger process at its current stage already has the potential to considerably cut biorefinery operating costs compared to, e.g., a state-of-the art SO_2_ pretreatment [49]. In the overall process cost of a scavenger process, the 2-naphthol additive ranked third after biomass and enzymes. However due to its enhancing effect on enzymatic conversion and the possibility to reduce enzyme dosages, the final process costs can be reduced.

It is further interesting to note that the acid-catalyzed pretreatment studies show a—sometimes dramatical—decrease of cellulose digestibility for high pretreatment severities [48, 50–52]. Nguyen et al. [50] specify that increasing severity to high values reduces enzymatic digestibility, but an explanation for this effect has not yet been presented in literature. We presume that severe lignin repolymerization played a role in decreasing the sugar yields and that acidic steam pretreatments of softwood can probably benefit from the addition of a carbocation scavenger, too. It has already been shown that the dilute acid pretreatment of spruce in stirred autoclaves can be enhanced by a carbocation scavenger [39].

Next to a high sugar yield from cellulose and hemicellulose, a value-added utilization of lignin can improve the economics of biorefining, in particular when high-lignin containing biomass like wood is used. Although intensive efforts have been made during the last decades, few breakthroughs have been achieved in developing high-value and marketable lignin products. Notably, the pretreatment significantly affects the physical and chemical characteristics of the resultant lignin and its utilization potential [5]. Lignin from common steam explosion and dilute acid pretreatment processes is however extensively repolymerized and therefore less valuable [5, 53]. In this respect, the resulting lignin from a scavenger process represents an interesting feedstock, owing to its lower and more uniform molecular weight, its low content in repolymerized aromatic C–C bonds, and an increased aromatic functionality due to the integration of the aromatic scavenger into the lignin structure [20].

## Conclusions

The study revealed that a carbocation scavenger can effectively be implemented in the steam pretreatment of lignocellulosic biomass. The approach can greatly enhance the enzymatic cellulose digestibility of softwood and the effect can complement the enhancing effect of the explosion well. The approach allows for remarkably high glucose yields in enzymatic hydrolysis without using harsh acids or removing lignin from the biomass. It therefore offers the possibility of obtaining high sugar yields from softwood in a simplified biorefinery concept.

Further development of the approach should focus on the optimization of the scavenger addition method and the implementation in a two-stage pretreatment. It is as well of interest to test the approach for biomass types like straw, corn stover or bagasse, which are already processed in commercial steam explosion plants at present.

## Additional file

**Additional file 1: Figure S1.** pH of pretreatment liquor after steam explosion pretreatments without additive and with 2-naphthol addition by mixing and impregnation. **Table S1.** Overview of biomass recovery and composition after pretreatment. **Table S2.** Overview of cellulose digestibility and sugar yields.

## Abbreviations

AIL: acid-insoluble lignin
ASL: acid-soluble lignin
FPU: filter paper units
HPLC: high-performance liquid chromatography
IAP: Industrieanlagen Planungsgesellschaft
NREL: National Renewable Energy Laboratory

## Footnotes

1 2-Naphthol solubility 43 g l^−1^, exponential interpolation of data from Moyle et al. [41].

2 Calculated with Wilson equation [54].
