# Supplementary material for: Pilot-scale steam explosion pretreatment with 2-naphthol to overcome high softwood recalcitrance
Source: Biotechnol Biofuels. 2017 May 18;10:130. doi: 10.1186/s13068-017-0816-y (PMC5437563; doi:10.1186/s13068-017-0816-y)
Supplement: Supplementary file 1 — Additional file 1: Figure S1. pH of pretreatment liquor after steam explosion pretreatments without additive and with 2-naphthol addition by mixing and impregnation. Table S1. Overview of biomass recovery and composition after pretreatment. Table S2. Overview of cellulose digestibility and sugar yields. [file 13068_2017_816_MOESM1_ESM.pdf]

Supporting information for:

Pilot scale steam explosion pretreatment with 2-naphthol to overcome high softwood recalcitrance

Thomas Pielhop,* Janick Amgarten, Michael H. Studer and Philipp Rudolf von Rohr*

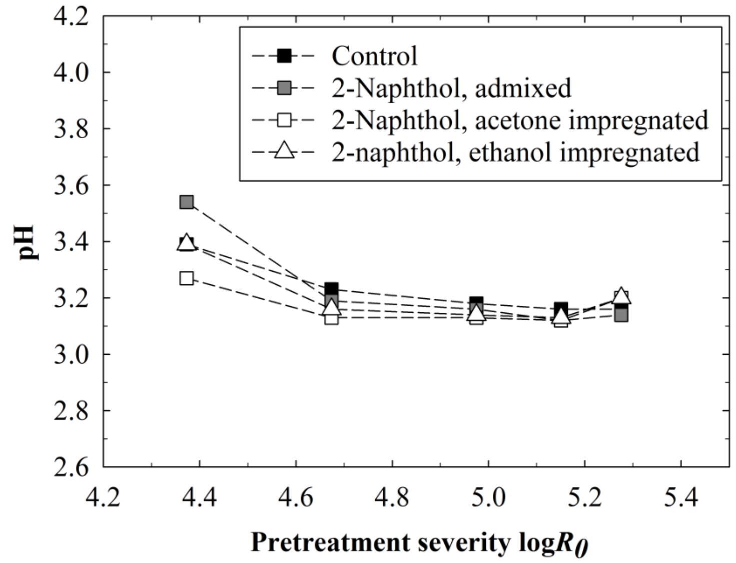

Fig. S1 pH of pretreatment liquor after steam explosion pretreatments without additive and with 2-naphthol addition by mixing and impregnation. Pretreatment conditions: T=235 °C, t=2.5–20 min, Δp explosion=30 bar, 1.5 kg wood chips, 35.36 g 2-naphthol.

Table S1 Overview of biomass recovery and composition after pretreatment.

2-Naphthol addition	T / °C	t / min	logR_0 / -	Δp expl. / bar	Biomass recovered / %	Cellulose recovered / %	Biomass composition			
							Cellulose / %	Mannan / %	AIL / %	ASL / %
-	235	2.5	4.4	30	89.5	86.7	35.9±1.0	11.1±2.4	40.8±0.2	6.1±0.1
-	235	5	4.7	30	90.4	93.3	45.0±1.0	6.5±1.2	45.4±0.7	7±0.1
-	235	10	5.0	30	77.4	85.3	40.8±0.0	3.3±0.2	52.5±0.3	8.7±0.4
-	235	15	5.2	30	75.7	85.3	41.7±0.4	2.3±0.8	53.6±0.3	8.8±0.3
-	235	20	5.3	30	75	86.4	42.7±0.2	2.5±0.4	53.2±0.3	7.6±1
Mixing	235	2.5	4.4	30	87.6	82.7	35.0±0.3	10.6±0.2	42.8±0.5	8.4±0.4
Mixing	235	5	4.7	30	95.3	93.3	39.4±1.1	5.2±1.1	47.2±0.3	12.3±0.5
Mixing	235	10	5.0	30	90.8	96.5	39.3±0.7	4.3±1.1	49.9±0.7	12.9±0.3
Mixing	235	15	5.2	30	87.1	98.6	41.9±0.8	-	54.7±1.8	13.3±0.4
Mixing	235	20	5.3	30	83.3	86.7	38.5±0.2	3±0.9	52.3±0.1	13.1±0.3
Mixing	235	2.5	4.4	2.5*	79.6	75.2	35.0±0.5	10.5±2.3	44.1±0.5	8±0.3
Mixing	235	5	4.7	2.5*	92.2	98.1	39.4±1.4	4.1±0.9	45.9±0.7	8.7±0.2
Mixing	235	10	5.0	2.5*	83.8	98.9	43.7±0.5	-	47.4±0.8	12.6±0.2
Mixing	235	15	5.2	2.5*	74.2	89	44.4±1.0	-	48.5±0.9	8.4±0.2
Mixing	235	20	5.3	2.5*	80.8	88.3	40.5±0.9	2.8±2.2	50.2±0.3	7.4±0.3
Acetone imp.	235	2.5	4.4	30	90.4	88.1	34.0±0.8	10.9±0.3	41.3±0.3	12.6±0.2
Acetone imp.	235	5	4.7	30	88.4	94.1	39.4±0.5	3.9±0.9	47±0.5	12.8±1.1
Acetone imp.	235	10	5.0	30	85.2	82.9	40.2±0.2	2.9±0.3	49.5±0.5	13.3±0.4
Acetone imp.	235	15	5.2	30	82.9	88.1	39.3±0.1	2.4±0.1	52.1±0.7	13.7±0.3
Acetone imp.	235	20	5.3	30	86.9	89.9	38.3±0.3	1.9±0.4	55.5±2.7	9.5±0.8
Ethanol imp.	235	2.5	4.4	30	87.1	103.2	43.9±0.9	8.2±0.5	45.6±0.8	7.9±0.5
Ethanol imp.	235	5	4.7	30	88.1	103.2	43.3±0.3	2.9±0.3	51.6±0.1	11.3±1.1
Ethanol imp.	235	10	5.0	30	89.2	99.1	41.2±0.4	2.1±0.2	57.1±0.5	9.3±0.3
Ethanol imp.	235	15	5.2	30	85.8	92.8	40.0±0.3	1.9±0.0	56.6±0.8	10.1±0.4
Ethanol imp.	235	20	5.3	30	83.3	93.4	40.0±0.2	2.2±0.6	56.8±1.0	9.9±0.6

* Experiments with a Δp of 2.5 bar are referred to as experiments "without explosion".

Table S2 Overview of cellulose digestibility and sugar yields. Yields are expressed as % of the raw biomass content. EH: yield in enzymatic hydrolysis (accounting for cellulose recovery from pretreatment); P: yield in pretreatment liquor; Mannose represents hemicellulosic sugars excluding glucose.

2-Naphthol addition	T / °C	t / min	logR_0 / -	Δp expl. / bar	FPU g^{-1} cellulose	Digestibility cellulose / %	Yields				
							Glucose, EH / %	Mannose, EH / %	Glucose, P / %	Mannose, P / %	Total sugar / %
					15	30.9±1.9	26.8	0			41.3
-	235	2.5	4.4	30	30	42.7±3.1	37.0	0	7.5±0.1	57.1±0.3	48.4
					60	57.3±5.7	49.7	0			57.1
					15	33.2±3.5	31.0	0			35.9
-	235	5	4.7	30	30	47.9±5.7	44.7	2.4	6.8±0.5	31.6±2.5	46.1
					60	62.3±4.3	58.1	2.9			62.7
					15	32.2±0.6	32.2	0			27.1
-	235	10	5.0	30	30	57.3±3.2	48.9	3.0	4.8±0.2	15.4±0.6	42.8
					60	88.0±3.7	75.1	7.3			62.2
					15	34.2±3.8	29.2	3			25.9
-	235	15	5.2	30	30	58.7±4.6	50.1	4.7	3.7±0.0	7±0.1	40.8
					60	87.4±1.7	74.6	7.8			58.8
					15	33.9±2.1	29.3	3			26.3
-	235	20	5.3	30	30	57.4±1.8	49.6	5.4	3.7±0.0	8.3±0.1	41.1
					60	90.2±3.9	77.9	6.7			61.1
					15	21.6±1	17.9	0			33.1
Mixing	235	2.5	4.4	30	30	37.6±0.3	31.1	0	6.6±0.0	52.3±0.1	42.2
					60	51.9±4.7	42.9	0			50.4
					15	47.4±2.4	44.2	3.6			47.9
Mixing	235	5	4.7	30	30	71.9±1.6	67.1	2.2	7.7	35.3	63.3
					60	92.5±4.5	86.3	13.4			80.1
					15	47.1±4.1	45.4	0.1			41.1
Mixing	235	10	5.0	30	30	77±4	75.3	3.8	5.8	32.9	61.1
					60	92.5±4.2	89.2	8.0			73.8
					15	49.9±5.9	49.2	4.3			41.8
Mixing	235	15	5.2	30	30	78.9±6.9	77.8	7.3	5.4	19.1	62.5
					60	90.4±4.2	89.2	11.4			71.7
					15	52.6±2.9	45.6	4.8			39.3
Mixing	235	20	5.3	30	30	75.6±1.6	65.5	6.9	4.3±0.1	10.7±0.1	53.7
					60	91.3±2.3	79.1	16.6			66.1
					15	27.9±4.4	19.8	1.5			29.8
Mixing	235	2.5	4.4	2.5*	30	28.3±1.8	20.1	4.5	4.8±0.1	37.1±0.1	30.9
					60	43.7±5.9	32.9	6.5			39.5
					15	29.7±4.2	29.1	0			34.3
Mixing	235	5	4.7	2.5*	30	36.6±3.7	35.9	1.6	7.7±0.0	35.3	39.5
					60	48.1±2.7	47.2	4.1			48.1
					15	44.7±5.1	44.2	0.3			39.6
Mixing	235	10	5.0	2.5*	30	46±2.9	45.5	0.6	5.8	32.9	40.6
					60	59.1±7.6	64.3	3.1			50.3
					15	59±3.1	52.5	1.5			43.5
Mixing	235	15	5.2	2.5*	30	63.7±6.6	56.7	1.2	4.5	19.1	46.3
					60	76.6±4.6	68.1	4.8			55.4
					15	60.3±1.4	53.3	2.8			42.0
Mixing	235	20	5.3	2.5*	30	73.1±2.4	64.6	2.9	3.1±0.1	6.9±0.0	49.9
					60	86.5±1	76.4	8.9			59.9
					15	47.9±3	39.7	0			44.0
Acetone imp.	235	2.5	4.4	30	30	73.7±0.3	61.1	0.5	4.9±0.0	15.8±0.0	59.0
					60	92.5±4.1	76.7	0			69.6
					15	71.2±3	67.0	1.1			55.0
Acetone imp.	235	5	4.7	30	30	93.3±1.8	87.9	1.7	4.9±0.0	15.8±0.0	69.5
					60	98.7±1.5	92.9	1.8			73.1
					15	83.1±4.1	76.8	1.9			60.1
Acetone imp.	235	10	5.0	30	30	93.3±1.2	86.3	2.4	4.9±0.0	15.8±0	66.8
					60	100.6±2.3	93.0	3.0			71.6
					15	80.9±5.1	71.3	2.3			54.4
Acetone imp.	235	15	5.2	30	30	94.2±0.3	83.0	2.7	3.7±0.0	6.1±0.0	62.6
					60	100.3±1.7	88.3	2.4			66.3
					15	94.7±1.3	85.20	3.1			62.4
Acetone imp.	235	20	5.3	30	30	96.3±1.1	86.60	3.1	2.5±0.0	2.8±0.0	63.5
					60	94.6±1.4	85.00	2.7			62.4

Ethanol imp.	235	2.5	4.4	30	15<br>30<br>60	57.3±2.1<br>78±1.5<br>83±1.5	59.1<br>80.5<br>85.7	0<br>0<br>0	5.4±0.0	33.9±0.1	40.9<br>55.7<br>59.3
Ethanol imp.	235	5	4.7	30	15<br>30<br>60	81.2±0.9<br>94.6±1.7<br>94.8±1.2	83.8<br>97.6<br>97.8	1.1<br>2.4<br>4.9	3.3±0.1	11.8±0.2	64.2<br>74.2<br>75.1
Ethanol imp.	235	10	5.0	30	15<br>30<br>60	92.6±0.4<br>95.9±2.5<br>95.7±2.1	91.80<br>95.00<br>94.80	2.5<br>2.8<br>3.6	2.9±0.0	3.9±0.1	67.4<br>69.8<br>69.9
Ethanol imp.	235	15	5.2	30	15<br>30<br>60	105.7±0.8<br>107.3±0.5<br>96.1±3	97.6<br>99.6<br>89.20	3.3<br>4.4<br>3.7	2.7±0.0	2.8±0.0	71.2<br>73.0<br>65.5
Ethanol imp.	235	20	5.3	30	15<br>30<br>60	83.7±1.2<br>88.2±2.2<br>91.2±2	78.2<br>82.4<br>85.1	3.1<br>2.4<br>3.9	2.9±0.0	4.4±0.0	58.4<br>61.8<br>63.4

* Experiments with a Δp of 2.5 bar are referred to as experiments “without explosion”.
